# US chiropractors’ attitudes, skills and use of evidence-based practice: A cross-sectional national survey

**DOI:** 10.1186/s12998-015-0060-0

**Published:** 2015-05-04

**Authors:** Michael J Schneider, Roni Evans, Mitchell Haas, Matthew Leach, Cheryl Hawk, Cynthia Long, Gregory D Cramer, Oakland Walters, Corrie Vihstadt, Lauren Terhorst

**Affiliations:** Department of Physical Therapy, School of Health and Rehabilitation Sciences, Clinical and Translational Science Institute, University of Pittsburgh, Pittsburgh, PA USA; Center for Spirituality and Healing, Integrative Health and Wellbeing Research Program, University of Minnesota, Minneapolis, MN USA; University of Western States, Portland, OR USA; School of Nursing and Midwifery, University of South Australia, Adelaide, SA Australia; Logan College of Chiropractic, Chesterfield, MO USA; Palmer Center for Chiropractic Research, Palmer College of Chiropractic, Davenport, IA USA; National University of Health Sciences, Lombard, IL USA; The Commonwealth Medical College, Scranton, PA USA; Department of Occupational Therapy, School of Health and Rehabilitation Sciences, University of Pittsburgh, Pittsburgh, PA USA

**Keywords:** Evidence-based medicine, Chiropractic, Complementary and alternative medicine, Survey research, Dissemination and implementation, Knowledge translation

## Abstract

**Background:**

Evidence based practice (EBP) is being increasingly utilized by health care professionals as a means of improving the quality of health care. The introduction of EBP principles into the chiropractic profession is a relatively recent phenomenon. There is currently a lack of information about the EBP literacy level of US chiropractors and the barriers/facilitators to the use of EBP in the chiropractic profession.

**Methods:**

A nationwide EBP survey of US chiropractors was administered online (Nov 2012-Mar 2013) utilizing a validated self-report instrument (EBASE) in which three sub-scores are reported: attitudes, skills and use. Means, medians, and frequency distributions for each of the sub-scores were generated. Descriptive statistics were used to analyze the demographic characteristics of the sample. Means and proportions were calculated for all of the responses to each of the questions in the survey.

**Results:**

A total of 1,314 US chiropractors completed the EBASE survey; the sample appeared to be representative of the US chiropractic profession. Respondents were predominantly white (94.3%), male (75%), 47 (+/− 11.6) years of age, and in practice for more than 10 years (60%). EBASE sub-score means (possible ranges) were: attitudes, 31.4 (8–40); skills, 44.3 (13–65); and use, 10.3 (0–24). Survey participants generally held favorable attitudes toward EBP, but reported less use of EBP. A minority of participants indicated that EBP coursework (17%) and critical thinking (29%) were a major part of their chiropractic education. The most commonly reported barrier to the use of EBP was “lack of time”. Almost 90% of the sample indicated that they were interested in improving their EBP skills.

**Conclusion:**

American chiropractors appear similar to chiropractors in other countries, and other health professionals regarding their favorable attitudes towards EBP, while expressing barriers related to EBP skills such as research relevance and lack of time. This suggests that the design of future EBP educational interventions should capitalize on the growing body of EBP implementation research developing in other health disciplines. This will likely include broadening the approach beyond a sole focus on EBP education, and taking a multilevel approach that also targets professional, organizational and health policy domains.

**Electronic supplementary material:**

The online version of this article (doi:10.1186/s12998-015-0060-0) contains supplementary material, which is available to authorized users.

## Background

Evidence-based practice (EBP) has been steadily advocated since the mid 1990’s [1,2] and has increasingly been adopted as a foundational framework for improving the quality of healthcare delivery systems [3,4]. Despite the growing awareness of EBP, there still remains a large gap between this appreciation of EBP and the actual uptake and application of EBP in clinical settings. This gap between knowledge and awareness of EBP - and actual clinical use of EBP - is found in almost all healthcare fields, including medicine, nursing, and physical therapy [5,6].

Over the past decade the chiropractic profession has also embraced EBP, as evidenced by new EBP educational programs at chiropractic institutions [7-11] and the creation of professional evidence-based chiropractic guidelines [12-16]. However, while the enthusiasm for EBP in chiropractic is encouraging, a key aspect of its success will be whether or not it translates to changes in clinical practice. These changes would include the reduced use of unsupported clinical tests and procedures, as well as increased emphasis on those with an evidence base [17].

Dissemination and implementation research provides the means to bridge the gap between EBP principles and their application in clinical practice. This is accomplished by examining the mechanisms by which research evidence is spread and affects change in healthcare providers’ attitudes and beliefs, and by evaluating strategies for changing healthcare professionals’ behaviors to include the uptake of evidence-based clinical practices [18].

Chiropractic is one of the largest complementary and alternative medicine (CAM) professions in the United States. An important step for addressing the EBP gap in the chiropractic profession is to first understand chiropractors’ attitudes and knowledge related to EBP, as well as the perceived barriers and facilitators to its application. Much of what is currently known is based on studies performed outside the United States. Walker et al. reported the results of an EBP survey showing that most of the 584 Australian chiropractors surveyed held positive attitudes towards EBP, thought EBP was useful, and were interested in improving their EBP skills [19]. However, despite their favorable inclination toward EBP, many Australian chiropractors stated they did not routinely use clinical practice guidelines. The three main barriers to the uptake of EBP identified in this study were: insufficient time, lack of generalizability of evidence to patient population, and inability to apply research findings to individual patients [19]. Similarly, researchers from Canada [20] and Great Britain [21] have found that accessibility to research, knowledge of how to access research, and critical appraisal skills are poor amongst chiropractors.

A study in a sub-specialty of 144 chiropractic orthopedists in the US also found favorable attitudes towards EBP, and a desire for EBP post-graduate continuing education [22]. The most frequently reported barriers to EBP in this study were lack of relevant clinical evidence and lack of time. Facilitators to EBP included internet and database access, online EBP educational materials, critical reviews of chiropractic research, and ability to download full-text articles. The findings of this study are limited however by its small and specialized sample; consequently, the factors associated with the uptake of EBP by the US chiropractic profession still remain poorly understood.

The purpose of this article is to describe the results of a cross-sectional survey of US chiropractors’ attitudes, skills and use of research evidence in clinical practice, as well as the barriers and facilitators to use of EBP.

## Methods

### Study design and setting

This was a cross-sectional survey conducted online between November 17, 2012 and March 5, 2013. The survey was administered electronically through the University of Pittsburgh (UPitt), Pittsburgh, Pennsylvania, using the UPitt web platform.

### Ethics

Ethical approval (PRO12060417) was obtained through the University of Pittsburgh’s (UPitt) institutional review board (IRB), which granted “exempt status” in June 2012. Informed consent was secured from all subjects on the homepage of the research website, prior to participation in the survey.

### Context

The *Distance Education Online Intervention for Evidence-Based Practice Literacy (DELIVER)* project is a two-phase NIH/NCCAM-funded study (R21 AT007547) designed to evaluate the effectiveness of an online EBP educational program on chiropractors’ attitudes, skill, and use of EBP. This cross-sectional survey comprised the first phase of the DELIVER study and served as a baseline measure of EBP literacy against which to analyze the effectiveness of an online EBP educational program (second phase).

### Participants & recruitment

The survey was open to all Doctors of Chiropractic (DCs) in the US who had internet access and a valid email address. A convenience sample of DCs were recruited primarily by emails forwarded to the membership rosters of several cooperating organizations including the following: American Chiropractic Association, Council on Chiropractic Guidelines and Practice Parameters, Congress of Chiropractic State Associations, Sacro Occipital Research Society International, Activator Methods, US ChiroDirectory, International College of Applied Kinesiology, the Pediatric Councils of the American Chiropractic Association and International Chiropractors Association.

These organizations provided email-forwarding services through their respective membership lists, which created a potential pool of over 30,000 DCs. The forwarded email message described a unique opportunity to participate in an online survey; participation was incentivized by offering participants the opportunity to enter a drawing to win an Apple iPad™. Recipients of the email were encouraged to forward the message on to their colleagues. Articles announcing the study and inviting readers to participate were published in two national chiropractic publications; Dynamic Chiropractic [23] and the Journal of American Chiropractic Association [24]. Another national chiropractic publication - The American Chiropractor - announced the study by sending an email blast to its national circulation of DCs.

### Questionnaire and outcomes

The **E**vidence-**B**ased Practice **A**ttitude and Utilization **S**urv**E**y (EBASE) is a self-administered instrument designed to measure CAM providers’ attitudes, skills and use of EBP [25]. The instrument has demonstrated good internal consistency, content validity, and acceptable test-retest reliability [26]. Minor modifications of a few EBASE items were required to ensure the language was appropriate for use with chiropractors [22]. These changes were made in consultation with the survey developer (ML) to ensure the structure and intent of the modified questions were not altered in any manner that would jeopardize the validity of the original survey. Only the demographics section (Part G) required major modifications to be relevant to the chiropractic profession.

Our modified version of the EBASE contains 75 items and is divided into 7 parts (Parts A-G); Parts A-F each address a different EBP construct, and Part G contains demographic items only. Each question within these parts allowed the participant 5 possible responses, which were rated numerically from 1 to 5 for Parts A and B, and from 0 to 4 for Part D. Although there are 7 parts to this survey, only 3 parts generate sub-scores: Parts A (Attitudes), B (Skill), and D (Use). The 4 remaining parts of the EBASE are not scored, including Part C (Training & Education), Part E (Barriers), Part F (Facilitators) and Part G (Demographics). The completion time of the online EBASE is approximately 10–12 minutes (see Additional file 1 for a copy of the modified EBASE survey and the scoring rubric used for calculating the three sub-scores).

### Survey administration and data collection

Interested DCs were invited to follow a link to a dedicated UPitt website where they could obtain detailed information about the study procedures and register for the study by submitting an email address. Participants were subsequently emailed a password to enter the survey site, an effort aimed at preventing multiple responses from the same individual. To encourage honest and transparent responses, anonymity was insured by assigning a unique identification number to each registered DC, which was used to identify the respondent’s survey data. As participants completed the survey, responses were captured through a secure data capturing feature/system, Web Data Xpress, an interface that allows for direct entry and storage of data within a designated SQL Server database. This method of data capture is resource-efficient and minimizes human error by avoiding the need for manual data entry.

### Data analysis

Data were analyzed using SPSS version 22 (SPSS Inc., Chicago, IL, USA). Since this was a cross-sectional survey we calculated descriptive statistics including response frequencies and means for each item in Parts A, B, D, E and F and response frequencies for Parts C and G. The attitudes, skills, and use sub-scores were calculated using the scoring rubric (see Additional file 1) developed with the original EBASE. This involves summing the first eight items of Parts A (response range 1–5; total score range of 8–40), all 13 of the items of Part B (response range 1–5; total score range of 13–65), and the first 6 items of Part D (response range 0–4; total score range of 0–24). Frequency distributions for the group sub-score means for Part A, B and D were also calculated. Higher sub-scores indicate higher self-reported levels of attitudes (Part A), skills (Part B) and use (Part D) of EBP.

## Results

### Sample Size

A total of 1,314 US chiropractors responded to the survey.

### Participant characteristics (Demographics and Education/Training)

Table 1 provides a summary of the frequencies of the demographic characteristics of the participants. The majority of the sample were male (75%), Caucasian (94%), practiced as sole proprietors (72%) and held a Bachelor’s or higher level graduate degree in addition to their chiropractic degree (>80%). The average age of our sample was 47 years (range: 24 to 85 years), and the mean number of years in practice was 17 years (range: 0 to 30 years or more).

**Table 1:** **Demographic characteristics of the 1,314 American chiropractors who completed the online evidence-based practice survey**

**Variable**	**Characteristic**	**n (%)**
**Gender**	Male	989 (75.3)
	Female	325 (24.7)
**Age**	Mean = 46.7 yrs (SD = 11.6); Range = 24-85 yrs	
**Race**	White	1239 (94.3)
	Black	13 (1.0 )
	Asian	33 (2.5 )
	Mixed Race/Other	29 (2.2 )
**Years since chiropractic graduation**	0-5	273 (20.8)
	6-10	146 (11.1)
	11-15	187 (14.2)
	16-20	159 (12.1)
	21-25	170 (12.9)
	26-29	144 (11.0)
	30 or more Mean = 17 yrs; Range = 0-30 or more yrs	235 (17.9)
**Highest education level**	High School	17 (1.3 )
	Associate’s Degree	214 (16.3)
	Bachelor’s Degree	821 (62.5)
	Master’s Degree	226 (17.2)
	Doctorate	36 (2.7 )
**Region of practice**	Midwest	380 (28.9)
	Northeast	287 (21.8)
	West	264 (20.1)
	Southeast	245 (18.6)
	Southwest	131 (10.0)
	Non-continental US	7 (0.5 )
**Geographic setting**	Suburban	629 (47.9)
	City	449 (34.2)
	Rural	236 (18.0)
**Patients seen daily**	0-10	367 (27.9)
	11-20	455 (34.6)
	21-30	259 (19.7)
	31-40	126 (9.6 )
	41-50	60 (4.6 )
	51 or more Median = 20/day; (IQR = 10-30) Range = 0-100/day	47 (3.6 )
**Focus of clinical practice**	**Musculoskeletal focus**	**869 (66.1)**
	Spine and extremities	742 (56.5)
	Spine	72 (5.5)
	Sports	55 (4.2)
	**Non-musculoskeletal focus**	**445 (33.9)**
	Family care	192 (14.6)
	Subluxation-based	114 (8.7)
	Wellness/Prevention	105 (8.0)
	Non-musculoskeletal	20 (1.5)
	Pediatrics	14 (1.1)
**Clinical role**	Sole Proprietor	946 (72.0)
	Partner or group practice	171 (13.0)
	Associate or employee	144 (11.0)
	Hospital-based practice	53 (4.0)
**Organizational membership**	Unaffiliated	722 (55.0)
	American Chiropractic Assoc. (ACA)	526 (40.0)
	International Chiropractors Assoc. (ICA)	66 (5.0)

SD = Standard Deviation. IQR = Interquartile Range. Yrs = Years.

Only a small minority of the sample indicated that the following topics were major parts of their chiropractic education: coursework about EBP (17%), applying research evidence to clinical practice (13%), and critical thinking/analysis (29%) (Table 2). Eleven percent of the sample indicated they never had any critical thinking/analysis included in their chiropractic education. Almost half the sample reported that they had never received any education/training on conducting systematic reviews (48%) or clinical research (42%).

**Table 2:** **Response frequency of Training/Education items (Part C of E-BASE)**

**PART C Item**	**None**	**Seminar (<1 day)**	**Short course (<1 week)**	**Minor part of chiropractic education**	**Major part of chiropractic education**	**Minor part of diplomate education**	**Major part of diplomate education**	**Academic diploma**	**Informal personal study**
Applying research evidence to clinical practice	8.1%	23.4%	5.7%	23.4%	13.1%	3.7%	3.9%	1.8%	17.0%
Critical thinking/critical analysis	10.8%	8.4%	5.3%	21.7%	29.0%	2.7%	3.8%	3.4%	14.9%
Evidence-based clinical practice/evidence-based chiropractic	4.8%	25.5%	5.5%	22.8%	17.0%	5.6%	4.9%	1.8%	12.1%
Conducting systematic reviews or meta-analysis	47.6%	6.3%	6.5%	21.8%	3.7%	1.9%	0.6%	1.2%	10.4%
Conducting clinical research	42.2%	6.3%	6.1%	26.5%	4.0%	2.4%	0.9%	1.8%	9.8%

These are responses to the question “Please indicate the highest level of training/education you have received in the following areas”.

### Descriptive results for parts A, B and D (Attitudes, Skills and Use)

**Figure 1:**
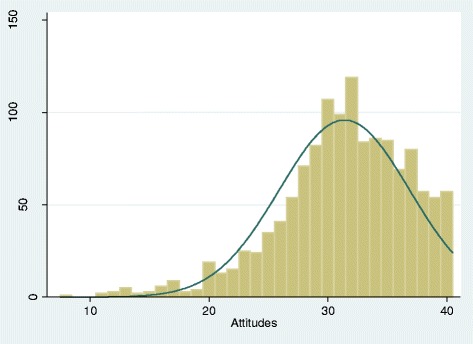
Frequency distribution of Attitudes sub-scores. The Y-axis indicates the number of survey participants and the X-axis indicates the Attitudes subscores. The mean sub-score was 31.4 (sd = 5.5) with a possible range of 8 to 40 (8 items scored 1–5). Median = 32.0 (IQR = 28-35).

Participants held a generally favorable attitude (Part A) toward EBP, with a mean attitude sub-score of 31.4 (range 8–40); while the frequency distribution was skewed to the left, the median sub-score (32.0) was close to the mean (Figure 1). The majority (>75%) of participants gave responses of “agree” or “strongly agree” to most questions (Table 3). There were two individual attitude related items with which a smaller proportion of the respondents agreed: 1) “EBP takes into account a patient’s preference for treatment” (42% agree/strongly agree); and 2) “EBP takes into account my clinical experience when making clinical decisions” (65% agree/strongly agree). It was also very interesting to note that the vast majority of our sample (89.5%) agreed or strongly agreed with the statement “I am interested in learning or improving the skills necessary to incorporate EBP into my practice”.

**Table 3:** **Response frequency and means of Attitudes toward EBP items (Part A of E-BASE)**

**Part A Items**	**Strongly Disagree(1)**	**Disagree(2)**	**Neutral(3)**	**Agree(4)**	**Strongly Agree(5)**	**Mean (Range=1-5)**
*I am interested in learning or improving the skills necessary to incorporate EBP into my practice	0.9%	2.1%	7.5%	42.8%	46.7%	4.3
*Evidence based practice (EBP) is necessary in the practice of chiropractic	2.1%	3.5%	9.1%	39.6%	45.7%	4.2
*Professional literature (i.e. journals & textbooks) and research findings are useful in my day-to-day practice	0.9%	3.4%	9.6%	53.4%	32.7%	4.1
*EBP improves the quality of my patient’s care	2.1%	4.4%	14.4%	43.2%	35.9%	4.1
*EBP assists me in making decisions about patient care	1.2%	3.7%	10.6%	48.6%	35.9%	4.1
Prioritizing EBP within chiropractic practice is fundamental to the advancement of the profession	2.4%	7.8%	13.3%	39.8%	36.7%	4.0
*EBP takes into account my clinical experience when making clinical decisions	2.3%	14.8%	17.7%	41.5%	23.7%	3.7
*The adoption of EBP places an unreasonable demand on my practice **[Note: Item is reverse coded]**	14.4%[5]	43.2[4]	29.1%[3]	10.6% [2]	2.7%[1]	3.6
*EBP takes into account a patient’s preference for treatment	5.3%	24.1%	28.5%	27.1%	15.0%	3.2
There is a lack of evidence from clinical trials to support most of the treatments I use in my practice	13.5%	42.2%	17.7%	22.6%	4.0%	2.6

*The sum of the 8 items with asterisks comprises the “Attitudes” sub-score, which ranges from 8-40. See Figure 1 for frequency distribution graph of attitudes sub-scores. These are responses to the question “On a scale ranging from strongly disagree to strongly agree, how would you rate your opinion on the following statements?”

**Figure 2:**
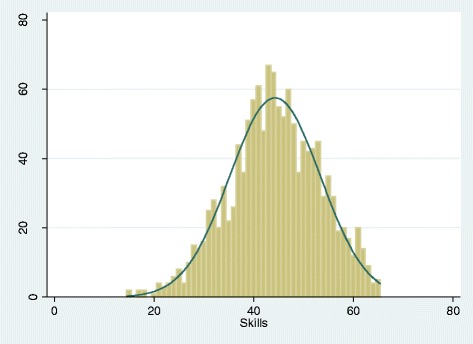
Frequency distribution of Skills sub-scores. The Y-axis indicates the number of survey participants and the X-axis indicates the Skills subscores. The mean sub-score was 44.3 (sd = 9.1) with a possible range of 13 to 65 (13 items scored 1–5). Median = 44.0 (IQR = 39-51).

For self-reported skills in EBP (Part B) the mean sub-score was 44.3 (range of 13–65) with a left skewed frequency distribution, and a median sub-score (44.0) similar to the mean (Figure 2). For the majority of skill items, more than half of respondents indicated a generally high level (‘4’ or ‘5’) of self-reported skill in EBP (Table 4); however nearly a third of respondents rated their skills in the mid-range (‘3’ on 1–5 scale) for 11 of the 13 skill items. The two skills rated poorest were: 1) “conducting clinical research” (66% of respondents) and 2) “conducting systematic reviews” (47% of respondents).

**Table 4:** **Response frequency and means of Skills in EBP items (Part B of E-BASE)**

**PART B Items**	**Poor (1)**	**(2)**	**(3)**	**(4)**	**Advanced(5)**	**Mean (Range=1-5)**
Identifying answerable clinical questions	0.3%	3.0%	18.6%	52.5%	25.6%	4.0
Identifying knowledge gaps in practice	0.4%	3.5%	29.5%	50.0%	16.7%	3.8
Locating professional literature	1.7%	8.6%	25.5%	38.7%	25.5%	3.8
Online database searching	3.7%	12.1%	25.3%	35.2%	23.7%	3.6
Retrieving evidence	3.0%	11.4%	28.5%	38.5%	18.6%	3.6
Critical appraisal of evidence	1.9%	10.6%	31.2%	42.4%	13.9%	3.6
Synthesis of research evidence	3.7%	15.8%	34.6%	36.6%	9.3%	3.3
Applying research evidence to patient cases	1.7%	8.0%	27.2%	48.9%	14.2%	3.7
Using findings from clinical research	1.5%	7.1%	29.1%	47.4%	14.9%	3.7
Sharing evidence with colleagues	4.8%	18.0%	30.6%	33.3%	13.3%	3.3
Using findings from systematic reviews	6.3%	19.2%	30.7%	34.0%	9.8%	3.2
Conducting systematic reviews	17.0%	29.9%	29.9%	18.4%	4.8%	2.6
Conducting clinical research	36.8%	29.5%	20.9%	10.3%	2.5%	2.1

The sum of all 13 items comprises the “skills” sub-score, which ranges from 13-65. See Figure 2 for frequency distribution graph of skills sub-scores. These are responses to the question “On a scale from 1 to 5, with 1 being poor and 5 being advanced, how would you rate your skills in the following areas”?

**Figure 3:**
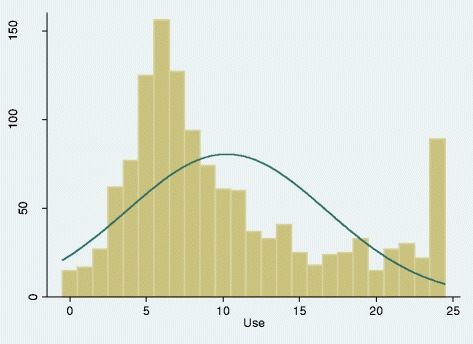
Frequency distribution of Use sub-scores. The Y-axis indicates the number of survey participants and the X-axis indicates the Use subscores. The mean sub-score was 10.3 (sd = 6.5) with a possible range of 0 to 24 (6 items scored 0–4). Median value = 8.0 (IQR = 6-14).

The mean sub-score for use of EBP (Part D) was 10.3 (range of 0–24). The frequency distribution was skewed to the right, with a median sub-score of 8.0 (Figure 3). While 36% reported not consulting magazines, laypersons or self-help books for clinical decision making in the previous month, 23% also reported not using research findings to change their clinical practice and 29% did not use an online database to search for practice-based literature or research findings. About 45% of the sample indicated that only a small, very small, or no proportion of their practice was based on clinical research evidence (Table 5).

**Table 5:** **Response frequency and means of Use of EBP items (Part D of E-BASE)**

**PART D Item**	**None (0)**	**Very small (1-25%) (1)**	**Small (26-50%) (2)**	**Moderate (51-75%) (3)**	**Large (76-99%) (4)**	**All (100%) (5)**	**Mean (Range=1-5)**
What percentage of your practice do you estimate is based on clinical research evidence (i.e. evidence from clinical trials)?	2.7%	21.2%	21.0%	32.3%	21.0%	1.8%	2.5
**PART D Items**	**0 times (0)**	**1-5 times (1)**	**6-10 times (2)**	**11-15 times (3)**	**16+ times (4)**		**Mean (Range=0-4)**
*I have read/reviewed professional literature (i.e. professional journals & textbooks) related to my practice	3.4%	41.9%	22.6%	8.7%	23.4%		2.1
*I have used an online search engine to search for practice related literature or research	7.9%	39.0%	23.5%	9.9%	19.7%		1.9
*I have read/reviewed clinical research findings related to my practice	7.8%	48.3%	17.4%	7.5%	19.0%		1.8
*I have used professional literature or research findings to assist my clinical decision making	11.0%	52.1%	14.8%	6.3%	15.8%		1.6
*I have used an online database to search for practice related literature or research	28.6%	36.5%	12.4%	6.4%	16.1%		1.4
*I have used professional literature or research findings to change my clinical Practice	23.2%	48.9%	11.3%	4.3%	12.3%		1.3
I have consulted a colleague or industry expert to assist my clinical decision making	22.5%	51.8%	13.5%	4.7%	7.5%		1.2
I have referred to magazines, layperson/self-help books, or non-government/non-education institution websites to assist my clinical decision making	35.6%	43.8%	11.1%	4.2%	5.3%		1.0

*The sum of the 6 items with asterisks comprises the “Use” sub-score, which ranges from 0-24. See Figure 3 for frequency distribution graph of the “use” sub-scores. These are responses to the question “Indicate how often you have performed the following activities over the last month”.

### Descriptive results for parts E and F (Barriers and Facilitators to EBP Uptake)

When presented with a list of potential barriers to EBP uptake (Part E), most (>75%) participants indicated that the majority of the factors were either “not a barrier” or a “minor barrier” (Table 6), with a few notable exceptions. Items rated as being moderate or major barriers to EBP uptake included: 1) ‘lack of time’ (48%); 2) ‘lack of clinical evidence about CAM’ (44%); 3), ‘lack of industry support’ (37%); and 4) ‘lack of incentive’ (36%). Approximately a quarter of respondents cited insufficient skills for interpreting (27%); locating (23%) and critically appraising research (24%); lack of colleague support for EBP (23%); and lack of relevance to chiropractic practice (24%).

**Table 6:** **Response frequency and means of Barriers to EBP uptake items (Part E of E-BASE)**

**PART E Items**	**Not a barrier (1)**	**Minor barrier (2)**	**Moderate barrier (3)**	**Major barrier (4)**	**Mean (Range = 1-4)**
Lack of time	19.2%	33.0%	34.1%	13.7%	2.4
Lack of clinical evidence in complementary and alternative medicine	18.9%	37.2%	32.2%	11.7%	2.4
Lack of industry support for EBP	31.4%	31.6%	26.3%	10.7%	2.2
Lack of incentive to participate in EBP	34.6%	29.2%	26.2%	10.0%	2.1
Insufficient skills for interpreting research	34.3%	38.7%	19.9%	7.1%	2.0
Insufficient skills for locating research	39.7%	37.3%	16.8%	6.2%	1.9
Insufficient skills to critically appraise/evaluate the literature	35.8%	39.8%	18.8%	5.6%	1.9
Lack of colleague support for EBP	44.1%	32.7%	17.0%	6.2%	1.9
Insufficient skills to apply research findings to clinical practice	39.9%	41.5%	15.8%	2.8%	1.8
Lack of relevance to chiropractic practice	47.1%	29.1%	17.8%	6.0%	1.8
Patient preference for treatment	41.5%	38.0%	16.8%	3.7%	1.8
Lack of interest in EBP	52.5%	30.5%	12.6%	4.4%	1.7
Lack of resources (i.e. access to a computer, the internet or online databases)	60.0%	26.8%	10.4%	2.8%	1.6

These are responses to the question “On a scale ranging from ‘not a barrier’ to ‘major barrier’, to what extent do the following factors prevent you from participating in EBP”?

In terms of the perceived usefulness of various factors in facilitating the uptake of EBP (Part F) in clinical practice, over 70% of respondents indicated all 10 items as either “moderately useful” or “very useful” (Table 7). The two items that received the greatest percentage of “very useful” responses were “access to the internet” (78%) and “access to free online databases” (70%). Items most frequently reported as “not useful” or “slightly useful” included: 1) “access to online tools to assist you to conduct your own critical appraisals of multiple research papers” (30%), and 2) “access to research rating tools that facilitate critical appraisal of single research papers” (26%).

**Table 7:** **Response frequency and means of Facilitators of EBP uptake items (Part F of E-BASE)**

**PART F Items**	**Not useful (1)**	**Slightly useful (2)**	**Moderately useful (3)**	**Very useful (4)**	**Mean (Range = 1-4)**
Access to the Internet in your workplace	3.4%	5.2%	13.5%	77.9%	3.7
Access to free online databases in the workplace, such as Cochrane and PubMed	2.0%	8.9%	18.9%	70.2%	3.6
Ability to download full-text / full-length journal articles	2.1%	11.5%	20.9%	65.5%	3.5
Access to online education materials related to evidence based practice	1.4%	9.3%	23.7%	65.6%	3.5
Access to critical reviews of research evidence relevant to your field (these are critical reviews of multiple research papers addressing a single topic)	1.8%	11.3%	31.4%	55.5%	3.4
Free access to online databases that usually require license fees, such as DynaMed and CINAHL	6.9%	15.1%	19.7%	58.3%	3.3
Access to critically appraised topics relevant to your field (these are critical appraisals of single research papers)	2.2%	15.6%	35.2%	47.0%	3.3
Access to tools used to assist the critical appraisal/evaluation of research evidence	3.4%	17.6%	36.7%	42.3%	3.2
Access to research rating tools that facilitate critical appraisal of single research papers	4.3%	21.9%	35.5%	38.3%	3.1
Access to online tools that assist you to conduct your own critical appraisals of multiple research papers related to a single topic	6.8%	22.9%	30.4%	39.9%	3.0

These are responses to the question “On a scale ranging from ‘not useful’ to ‘very useful’, to what extent would the following strategies assist you in participating in EBP”?

## Discussion

This is one of the largest studies to examine chiropractors’ perspectives relative to EBP, and to our knowledge, the first national survey to be conducted in the United States. One other EBP study was performed in the US, however the sample was limited to mid-western chiropractors with advanced training in orthopedics [22]. Despite the large absolute sample size of our survey (n = 1,314), it represents only a small relative cross-sectional sample of the American chiropractic profession (n = 60,000) [27-29]. However, the demographic characteristics of our sample (Table 1) are strikingly similar to those reported by three National Board of Chiropractic Examiners’ Job Analysis Reports [27-29]. This provides support for the generalizability of our survey results and makes us more confident that we have obtained a representative sample of US chiropractors.

The results suggest that our respondents generally have positive attitudes about evidence-based practice and a high level of self-reported skills in EBP, but only a modest level of EBP uptake in their clinical practices. These results are relatively similar to those reported from a recent EBP survey of Australian chiropractors [19] and are consistent with the observation that passive diffusion of knowledge does not automatically translate into clinical implementation [6]. Further, it emphasizes the need for high quality EBP continuing education programs to meet the needs of the chiropractic profession.

Our participants reported generally positive attitudes toward EBP, with most agreeing that EBP is important for improving practice, patient care, and advancing the profession. Noteworthy was that nearly a third strongly agreed that there is a ‘lack of evidence from clinical trials to support most of the treatments I use in my practice’. Similarly, only 42% agreed or strongly agreed that ‘EBP takes into account a patient’s preference for treatment’. These findings suggest that the basic principles of EBP may be misunderstood by DCs given the original definition of EBP clearly states that clinical expertise, patient values and best available research evidence are all integral components of evidence-based practice [1]. However, these opinions might also reflect what has become a growing recognition across healthcare fields; that clinical research needs to become more patient-oriented, pragmatic and generalizable to “real life” clinical practice [30].

Our sample of DCs reported that their poorest EBP skills were in conducting clinical research and/or systematic reviews; given that this survey was of practicing DCs without academic or research affiliation, this is not surprising. Distinctions have been drawn between the expectation for practitioners to be ‘consumers’ who ‘use’ research rather than ‘manufacturers’ who ‘produce’ research [31]; future studies should take this into consideration by ensuring that data collection instruments reflect this thinking.

Most of our sample reported above average skills in EBP, particularly in relation to identifying answerable clinical questions, identifying knowledge gaps in practice, locating professional literature and online database searching. However, nearly a third of respondents rated themselves only in the mid-range on nearly all of the EBP skill items. Some of these skills included the ability to synthesize research evidence, sharing evidence with colleagues, and using the findings from systematic reviews. Interestingly, while almost two-thirds reported above-average to advanced skills in using findings from clinical research, less than half reported the same level of skill in using findings from systematic reviews. This suggests that DCs have a limited understanding of the value and availability of systematic reviews, which is problem shared by many health professionals [32].

The introduction of EBP into the curricula of US chiropractic colleges is a relatively new phenomenon that has largely occurred over the past decade. The National Center for Complementary and Integrative Health (formerly NCCAM) has provided funding through its R25 mechanism to nine CAM colleges - four of them with chiropractic education programs - to develop institutional programs focused on teaching EBP. An overarching goal of these R25 research education grants was to provide CAM faculty and students with the skills they need to apply a rigorous evidence-based perspective to their training and practice. Adding research literacy and EBP competencies to the curricula at these CAM colleges has led to changes in their institutional cultures, such as increased faculty use of EBP case studies in the classroom and student-led research/journal clubs [33-35]. However, with approximately two thirds of our sample receiving their chiropractic training 11 to 30 years ago, it is likely that many of our participants never received what would now be considered foundational training in EBP.

Additionally, our results suggest that educational emphasis should be focused on improving the skills of appraising and applying research evidence in clinical practice. This needs to be done in a way that provides clinicians with ‘real life’ clinical examples, in order to overcome the barriers of lack of interest or clinical relevance to chiropractic practice. This issue was addressed in the second phase of our project, which explores the effectiveness of online EBP educational modules and “booster exercises” that incorporate clinical examples relevant to chiropractors. Results of the second phase of this project will be reported in a future publication.

The results of this survey also indicate that there are serious gaps in the uptake of research evidence into chiropractic practice, with nearly half reporting only a very small proportion of what they do in their clinical practice is based on research evidence. DCs appear to have challenges with performing online searches of the literature and interpreting the results of systematic reviews. Although most DCs in our sample reported they had above average skills in locating literature online, they also indicated that they did not engage in the uptake of EBP on a frequent basis (> six times a month). This apparent contradiction may be associated with the issues of time and lack of evidence, as discussed in the next paragraph. However, almost 90% of our sample indicated that they were interested in learning or improving the skills necessary to incorporate EBP into their practices (Table 3). Educational interventions and strategies are more likely to be successful if they are informed by known barriers and facilitators [36-39].

On the whole, most DCs indicated there were few barriers to their uptake of EBP, which is consistent with their generally positive attitudes toward EBP. It is worth noting however, that almost half of DCs indicated that the two biggest barriers to EBP uptake were ‘lack of time’ and ‘lack of clinical evidence in CAM’. A sizeable proportion (a quarter to one third) also cited: ‘lack of industry support’; ‘lack of incentive’; ‘insufficient skills for interpreting research’; ‘locating and critically appraising research’; ‘lack of colleague support for EBP’; and ‘lack of relevance to chiropractic practice’. Many of the barriers identified in this study are similar to those found for chiropractors in Australia [19], Canada [20] and Great Britain [21] as well as a sub-specialty of chiropractic orthopedists in the US [22]. Interestingly, many of the same barriers are encountered in the medical and nursing professions [5,6], leading us to conclude that the challenges facing the chiropractic profession in implementing EBP are not unique.

Interestingly, very few DCs indicated that computer, internet, or database access were barriers to their uptake of EBP. Coupled with our sample’s perceived usefulness of all of the listed facilitator items, these findings underscore the importance of providing clinicians with training in EBP skills, particularly through online resources. Our findings also suggest a need for greater support from professional organizations to facilitate collegial support of EBP, as well as better collaboration between scientists and practitioners in the design of clinically applicable research. Indeed, while educational strategies are an important part of narrowing the gap between science and practice for chiropractic and other health disciplines, they will likely be insufficient on their own to accomplish true change. Comprehensive and multi-faceted approaches that take into account all the relevant levels affecting EBP, including professional, managerial, organizational and health systems, will likely be needed to integrate research into practice [41].

There were several limitations to this study. The first is inherent to any type of survey design, which is reliance on self-reporting. For example, the ‘skills’ sub-score was based upon the participants’ self-perceived level of skill; we did not directly test a participant’s knowledge or skills in EBP. It would be useful in future studies to correlate an actual “grade” from tests or quizzes of EBP knowledge with the self-reported survey data. Another inherent limitation is selection bias; it is possible that the ‘attitudes’ sub-scores were skewed toward higher values because participants were already in favor of an evidence-based practice paradigm prior to commencing the survey.

Although we had a relatively large number of survey responders (n = 1,314), this number represents only a small proportion of the approximately 60,000 licensed chiropractors in the US. We made some minor modifications in the original EBASE questionnaire, chiefly to use the word “chiropractic” in certain questions. We do not believe these minor changes altered the intrinsic properties of the EBASE, however we did not formally conduct a psychometric evaluation of this modified version.

## Conclusion

The results of this survey have provided new insights into the attitudes, skills and use of EBP among US chiropractors. The information gained from this survey will be most helpful in informing the design of future educational interventions for chiropractors to improve their level of EBP literacy and use of evidence in clinical practice. Overall, American chiropractors appear very similar to chiropractors in other countries, as well as other health professionals in terms of their favorable attitudes towards EBP, while expressing limitations and barriers related to EBP skills, research relevance, and lack of time. This suggests that the design of future EBP interventions for chiropractic should capitalize on the growing body of EBP implementation research evidence developing in other health disciplines. This will likely include broadening the approach beyond a sole focus on EBP education, and taking a multilevel approach that also targets professional, organizational and health policy domains.

## Additional file

Additional file 1:
**EBASE Questionnaire and Scoring Rubric.**

## Abbreviations

ACA: American Chiropractic Association
CAM: Complementary and Alternative Medicine
DELIVER: Distance Education Online Intervention for Evidence-Based Practice Literacy
DC: Doctor of Chiropractic
EBP: Evidence-Based Practice
EBASE: Evidence-Based Practice Attitude and Utilization Survey
UPitt: University of Pittsburgh
NCCAM: National Center for Complementary and Alternative Medicine
