# Supplementary material for: US chiropractors’ attitudes, skills and use of evidence-based practice: A cross-sectional national survey
Source: Chiropr Man Therap. 2015 May 4;23:16. doi: 10.1186/s12998-015-0060-0 (PMC4422535; doi:10.1186/s12998-015-0060-0)
Supplement: Additional file 1: — EBASE Questionnaire and Scoring Rubric. [file 12998_2015_60_MOESM1_ESM.docx]

**ADDITIONAL FILE 1**

**EBASE Questionnaire and Scoring Rubric**

(modified for chiropractic profession)

Thank you for agreeing to complete this important survey. Your support in completing this questionnaire is valued and greatly appreciated. The following questionnaire aims to identify the use, opinion, skills and training in evidence-based practice (EBP) for doctors of chiropractic.

This questionnaire is estimated to take approximately 10 minutes to complete. For most questions, please indicate how you feel at the time of completing the survey by clicking the circle next to your best selection (please try to avoid answering ‘neutral’ unless you really are uncertain).

**PART A**

**On a scale ranging from strongly disagree to strongly agree, how would you rate your opinion on the following statements?** (please select one best answer per category)

**1. Evidence-based practice is necessary in the practice of chiropractic**

 Strongly Disagree  Disagree  Neutral  Agree  Strongly Agree

**2. Professional literature (ie: journals & textbooks) and research findings are useful in my day-to-day practice**

 Strongly Disagree  Disagree  Neutral  Agree  Strongly Agree

**3. I am interested in learning or improving the skills necessary to incorporate EBP into my practice**

 Strongly Disagree  Disagree  Neutral  Agree  Strongly Agree

**4. Evidence-based practice improves the quality of my patient’s care**

 Strongly Disagree  Disagree  Neutral  Agree  Strongly Agree

**5. Evidence-based practice assists me in making decisions about patient care**

 Strongly Disagree  Disagree  Neutral  Agree  Strongly Agree

**6. Evidence-based practice takes into account my clinical experience when making clinical decisions**

 Strongly Disagree  Disagree  Neutral  Agree  Strongly Agree

**7. Evidence-based practice takes into account a patient’s preference for treatment**

 Strongly Disagree  Disagree  Neutral  Agree  Strongly Agree

**8. The adoption of evidence-based practice places an unreasonable demand on my practice**

 Strongly Disagree  Disagree  Neutral  Agree  Strongly Agree

**9. There is a lack of evidence from clinical trials to support most of the treatments I use in my practice**

 Strongly Disagree  Disagree  Neutral  Agree  Strongly Agree

**10. Prioritizing evidence-based practice within chiropractic practice is fundamental to the advancement of the profession**

 Strongly Disagree  Disagree  Neutral  Agree  Strongly Agree

**PART B**

**On a scale from 1 to 5, with 1 being poor and 5 being advanced, how would you rate your skills in the following areas?** (please select one per skill area)

**11. Identifying knowledge gaps in practice**

 1 (poor)  2  3  4  5 (advanced)

**12. Identifying answerable clinical questions**

 1 (poor)  2  3  4  5 (advanced)

**13. Locating professional literature (ie: journal articles)**

 1 (poor)  2  3  4  5 (advanced)

**14. Online database searching (ie: MEDLINE)**

 1 (poor)  2  3  4  5 (advanced)

**15. Retrieving evidence**

 1 (poor)  2  3  4  5 (advanced)

**16. Critical appraisal of evidence**

 1 (poor)  2  3  4  5 (advanced)

**17. Synthesis of research evidence**

 1 (poor)  2  3  4  5 (advanced)

**18. Applying research evidence to patient cases**

 1 (poor)  2  3  4  5 (advanced)

**19. Sharing evidence with colleagues**

 1 (poor)  2  3  4  5 (advanced)

**20. Conducting clinical research (ie: clinical trials)**

 1 (poor)  2  3  4  5 (advanced)

**21. Using findings from clinical research**

 1 (poor)  2  3  4  5 (advanced)

**22. Conducting systematic reviews**

 1 (poor)  2  3  4  5 (advanced)

**23. Using findings from systematic reviews**

 1 (poor)  2  3  4  5 (advanced)

**PART C**

**Please indicate the highest level of training/education you have received in the following areas** (please select one best answer per category). **If you tick ‘other’, please write down your highest level of training/education in the space provided.**

**24. Evidence-based clinical practice / Evidence-based chiropractic**

 None  Major part of chiropractic education

 Seminar (< 1 day)  Minor part of diplomate education

 Short course (< 1 week)  Major part of diplomate education

 Specific course (>1 week)  Academic diploma

 Minor part of chiropractic education  Informal personal study (i.e. books,

internet, journals)

 Other (please specify) ____________________________________________________

**25. Applying research evidence to clinical practice**

 None  Major part of chiropractic education

 Seminar (< 1 day)  Minor part of diplomate education

 Short course (< 1 week)  Major part of diplomate education

 Specific course (>1 week)  Academic diploma

 Minor part of chiropractic education  Informal personal study (i.e. books,

internet, journals)

 Other (please specify) ____________________________________________________

**26. Conducting clinical research (ie: clinical trials)**

Part C

 None  Major part of chiropractic education

 Seminar (< 1 day)  Minor part of diplomate education

 Short course (< 1 week)  Major part of diplomate education

 Specific course (>1 week)  Academic diploma

 Minor part of chiropractic education  Informal personal study (i.e. books,

internet, journals)

 Other (please specify) ____________________________________________________

**27. Conducting systematic reviews or meta-analysis(ie: statistical analysis of data combined from two or more studies)**

 None  Major part of chiropractic education

 Seminar (< 1 day)  Minor part of diplomate education

 Short course (< 1 week)  Major part of diplomate education

 Specific course (>1 week)  Academic diploma

 Minor part of chiropractic education  Informal personal study (i.e. books,

internet, journals)

 Other (please specify) ____________________________________________________

**28. Critical thinking / critical analysis**

 None  Major part of chiropractic education

 Seminar (< 1 day)  Minor part of diplomate education

 Short course (< 1 week)  Major part of diplomate education

 Specific course (>1 week)  Academic diploma

 Minor part of chiropractic education  Informal personal study (i.e. books,

internet, journals)

 Other (please specify) ____________________________________________________

**PART D**

**Please indicate how often you have performed the following activities over the last month** (please select one best answer per category).

**29. I have read/reviewed professional literature (ie: professional journals & textbooks) related to my practice**

 0 articles  1-5 articles  6-10 articles  11-15 articles  16+ articles

**30. I have read/reviewed clinical research findings related to my practice**

 0 articles  1-5 articles  6-10 articles  11-15 articles  16+ articles

**31. I have used professional literature or research findings to assist my clinical decision-making**

 Never  1-5 times  6-10 times  11-15 times  16+ times

**32. I have used professional literature or research findings to change my clinical practice**

 Never  1-5 times  6-10 times  11-15 times  16+ times

**33. I have used an online database (ie: CINAHL, MEDLINE) to search for practice related literature or research**

 Never  1-5 times  6-10 times  11-15 times  16+ times

**34. I have used an online search engine (ie: Google) to search for practice related literature or research**

 Never  1-5 times  6-10 times  11-15 times  16+ times

**35. I have consulted a colleague or industry expert to assist my clinical decision-making**

 Never  1-5 times  6-10 times  11-15 times  16+ times

**36. I have referred to magazines, layperson/self help books, or non-government/**

**non-education institution websites to assist my clinical decision-making**

 Never  1-5 times  6-10 times  11-15 times  16+ times

**37. What percentage of your practice do you estimate is based on clinical research evidence (ie: evidence from clinical trials)?** (please tick only one response)

 None (0%)  Moderate (51-75%)

 Very small proportion (1-25%)  Large proportion (76-99%)

 Small proportion (26-50%)  All (100%)

**38. When you are making clinical decisions, in what order do the following sources of information inform the basis of your decision? Please rank the items from 1 to 10, with 1 being the most frequently used source of information, to 10, being the least frequently used source of information:**

 Published clinical evidence (ie: clinical trials)

 Published experimental/laboratory evidence (ie: animal or test tube studies)

 Traditional knowledge

 Consulting fellow practitioners or experts

 Personal intuition

 Trial and error

 Textbooks

 Clinical practice guidelines

 Patient preference

 Personal preference

**PART E**

**On a scale ranging from ‘not a barrier’ to ‘major barrier’, to what extent do the following factors prevent you from participating in evidence-based practice?**

**39. Lack of time**

 Not a barrier  A minor barrier  A moderate barrier  A major barrier

**40. Lack of resources (ie: access to a computer, the internet or online databases)**

 Not a barrier  A minor barrier  A moderate barrier  A major barrier

**41. Lack of clinical evidence in complementary and alternative medicine**

 Not a barrier  A minor barrier  A moderate barrier  A major barrier

**42. Insufficient skills for locating research**

 Not a barrier  A minor barrier  A moderate barrier  A major barrier

**43. Insufficient skills for interpreting research**

 Not a barrier  A minor barrier  A moderate barrier  A major barrier

**44. Insufficient skills to critically appraise / evaluate the literature**

 Not a barrier  A minor barrier  A moderate barrier  A major barrier

**45. Insufficient skills to apply research findings to clinical practice**

 Not a barrier  A minor barrier  A moderate barrier  A major barrier

**46. Lack of incentive to participate in evidence-based practice**

 Not a barrier  A minor barrier  A moderate barrier  A major barrier

**47. Lack of interest in evidence-based practice**

 Not a barrier  A minor barrier  A moderate barrier  A major barrier

**48. Lack of relevance to chiropractic practice**

 Not a barrier  A minor barrier  A moderate barrier  A major barrier

**49. Lack of colleague support for evidence-based practice**

 Not a barrier  A minor barrier  A moderate barrier  A major barrier

**50. Lack of industry support for evidence-based practice**

 Not a barrier  A minor barrier  A moderate barrier  A major barrier

**51. Patient preference for treatment**

 Not a barrier  A minor barrier  A moderate barrier  A major barrier

Part E

**PART F**

**On a scale ranging from ‘not useful’ to ‘very useful’, to what extent would the following strategies assist you in participating in evidence-based practice?**

**52. Access to the Internet in your workplace**

 Not useful  Slightly useful  Moderately useful  Very useful

**53. Access to free online databases in the workplace, such as Cochrane and Pubmed**

 Not useful  Slightly useful  Moderately useful  Very useful

**54. Free access to online databases that usually require license fees, such as DynaMed and CINAHL**

 Not useful  Slightly useful  Moderately useful  Very useful

**55. Ability to download full-text / full-length journal articles**

 Not useful  Slightly useful  Moderately useful  Very useful

**56. Access to online education materials related to evidence-based practice**

 Not useful  Slightly useful  Moderately useful  Very useful

**57. Access to tools used to assist the critical appraisal / evaluation of research evidence**

 Not useful  Slightly useful  Moderately useful  Very useful

**58. Access to critically appraised topics relevant to your field (these are critical appraisals of single research papers)**

 Not useful  Slightly useful  Moderately useful  Very useful

**59. Access to critical reviews of research evidence relevant to your field (these are critical reviews of multiple research papers addressing a single topic)**

 Not useful  Slightly useful  Moderately useful  Very useful

**60. Access to research rating tools that facilitate critical appraisal of single research papers**

 Not useful  Slightly useful  Moderately useful  Very useful

**61. Access to online tools that assist you to conduct your own critical appraisals of multiple research papers related to a single topic**

 Not useful  Slightly useful  Moderately useful  Very useful

**PART G : Demographic Questions**

**62.** **Date of Birth**: _____ [use drop down menus for day/month/year]

**63.** **Gender**: □Male □Female

**64. Do you consider yourself to be Hispanic or Latino?** □ Yes □ No

**65. What race do you consider yourself to be**? [check all that apply]

□American Indian/Alaskan Native □ Asian

□ Native Hawaiian/Pacific Islander □ White/Caucasian

□ Black/African American □ Mixed race

**66. Chiropractic college of graduation:**

□ Canadian Memorial □Cleveland (KC) □ Cleveland (LA)

□ Life West □ Life (Marietta) □ Logan

□ National □ New York □ Northwestern

□ Palmer (Davenport) □ Palmer (Florida) □ Palmer (West)

□Parker □ Sherman □ Southern California

□ Texas □ Bridgeport □ Western States

□ D’Youville □ Other (please specify): ________ [fillable field]

**67. Year of chiropractic college graduation**: ____

[use drop down menus for day/month/year]

**68. Your highest level of education besides your DC degree:** [check one box only]

□ High School □ Associate’s Degree □ Bachelor’s Degree

□ Master’s Degree □ Doctorate (PhD) Degree

**69. Do you hold any post-graduate diplomate status through a specialty board, council, academy, or association?** [check one box only]

□ Have a diplomate □ Currently working on diplomate □ None

**70. In which state is your primary practice?** _____

[use drop down menu with all 50 states; District of Columbia, Puerto Rico, US Virgin Islands]

**71. In which type of geographic setting do you practice**? [check one box only]

□ Urban □ Suburban □ Rural

**72. What is your role in the clinical setting in which you practice?**

[check one box only]

□ Associate/employee □ Sole proprietor/Solo practice

□ Sole proprietor within group □ Owner/Partner multi-disciplinary practice

□ Partnership within group □ Hospital

**73. What is the average number of patient visits that you personally**

**(not clinic) see weekly?** ____ [fillable field]

**74. What is the main focus of your chiropractic care?** [check one box only]

□ Pediatrics □ Family Care □ Wellness/Prevention □ Sports □ Non-musculoskeletal care □ Spine

□ General musculo-skeletal care (spine and extremities) □ Subluxation-based

**75. Please indicate your organizational status.** [check only one box]

□ ACA member □ ICA member □ No membership

**Rubric for calculating EBASE Sub-scores**

**PART A: Attitude Sub-score**

***Summary of part A***

- *Number of items*: 8 items (omit items #9 and #10)
- *Response type*: 5 point Likert scale (strongly disagree to strongly agree)
- *Score per item*: 1 (strongly disagree) to 5 (strongly agree)
- *Range of possible total scores*: 8-40

***Scoring***

1. Score items #1 through to #7 as outlined above

2. Reverse score item #8

3. Sum all scores for items #1 through to #8

4. Scores can be interpreted using the following quartiles:

a. Q1=8.0-16.0 (predominantly strongly disagree to disagree)

b. Q2=16.1-24.0 (predominantly neutral to disagree)

c. Q3=24.1-31.9 (predominantly neutral to agree)

d. Q4=32.0-40.0 (predominantly agree to strongly agree)

**PART B: Skill Sub-score**

***Summary of part B***

- *Number of items*: 13 items
- *Response type*: 5 point skill scale (poor skill to advanced skill)
- *Score per item*: 1 (poor skill) to 5 (advanced skill)
- *Range of possible total scores*: 13-65

***Scoring***

1. Score items #11 through to #23 as outlined above

2. Sum all scores for items #11 through to #23

3. Scores can be interpreted using the following quartiles:

a. Q1=13.0-26.0 (predominantly poor to somewhat poor)

b. Q2=26.1-39.0 (predominantly somewhat poor to average)

c. Q3=39.1-51.9 (predominantly average to somewhat advanced)

d. Q4=52.0-65.0 (predominantly somewhat advanced to advanced)

**PART D: Use Sub-score**

***Summary of part D***

- *Number of items*: 6 items (omit items #35 and #36)
- *Response type*: 5 point use scale (zero/never to 16+ times)
- *Score per item*: 0 (zero/never) to 4 (16+ times)
- *Range of possible total scores*: 0-24

***Scoring***

1. Score items #29 through to #34 as outlined above

2. Sum all scores for items #29 through to #34
